# In the Aftermath of Acute Hospitalization for Traumatic Brain Injury: Factors Associated with the Direct Pathway into Specialized Rehabilitation

**DOI:** 10.3390/jcm10163577

**Published:** 2021-08-14

**Authors:** Cathrine Tverdal, Nada Andelic, Eirik Helseth, Cathrine Brunborg, Pål Rønning, Torgeir Hellstrøm, Cecilie Røe, Mads Aarhus

**Affiliations:** 1 Department of Neurosurgery, Oslo University Hospital, 0424 Oslo, Norway; ehelseth@ous-hf.no (E.H.); paroen@ous-hf.no (P.R.); madaar@ous-hf.no (M.A.); 2 Institute of Clinical Medicine, Faculty of Medicine, University of Oslo, 0318 Oslo, Norway; cecilie.roe@medisin.uio.no; 3 Department of Physical Medicine and Rehabilitation, Oslo University Hospital, 0424 Oslo, Norway; uxhetz@ous-hf.no; 4 Research Centre for Habilitation and Rehabilitation Models and Services (CHARM), Institute of Health and Society, Faculty of Medicine, University of Oslo, 0373 Oslo, Norway; 5 Oslo Centre for Biostatistics and Epidemiology, Research Support Services, Oslo University Hospital, 0317 Oslo, Norway; uxbruc@ous-hf.no

**Keywords:** traumatic brain injury, rehabilitation, care pathway, predictors, trauma hospital

## Abstract

Previous research has demonstrated that early initiation of rehabilitation and direct care pathways improve outcomes for patients with severe traumatic brain injury (TBI). Despite this knowledge, there is a concern that a number of patients are still not included in the direct care pathway. The study aim was to provide an updated overview of discharge to rehabilitation following acute care and identify factors associated with the direct pathway. We analyzed data from the Oslo TBI Registry—Neurosurgery over a five-year period (2015–2019) and included 1724 adults with intracranial injuries. We described the patient population and applied multivariable logistic regression to investigate factors associated with the probability of entering the direct pathway. In total, 289 patients followed the direct pathway. For patients with moderate–severe TBI, the proportion increased from 22% to 35% during the study period. Significant predictors were younger age, low preinjury comorbidities, moderate–severe TBI and disability due to TBI at the time of discharge. In patients aged 18–29 years, 53% followed the direct pathway, in contrast to 10% of patients aged 65–79 years (moderate–severe TBI). This study highlights the need for further emphasis on entering the direct pathway to rehabilitation, particularly for patients aged >64 years.

## 1. Introduction

The physical, cognitive and emotional consequences of traumatic brain injury (TBI) may have a substantial negative impact on daily life functioning and quality of life [1,2]. The goal of TBI rehabilitation is to maximize the final outcome and preferably restore the preinjury functional level. Specialized TBI rehabilitation is provided by multidisciplinary teams working in a coordinated effort. Ideally, such rehabilitation should start as soon as the patient is in a medically stable phase and would be part of an uninterrupted chain of treatments (direct pathway). Studies have shown improved outcomes for patients who receive more intense and early initiation of rehabilitation and follow a direct pathway into rehabilitation [3,4,5,6]. However, this goal may not be achievable in all patients. It appears that only 41–50% of patients with severe TBI are referred directly from regional acute care to brain injury rehabilitation units [7,8,9]. Furthermore, direct pathway interruptions may have a negative effect on functional outcomes for individuals with severe TBI [8,10].

Clinical factors positively associated with access to TBI rehabilitation include more severe injury (moderate to severe TBI), intracranial and extracranial surgery, length of stay and impaired function [11,12]. Studies determining the predictive value of demographics demonstrate that younger age is associated with discharge to rehabilitation and that the association of sex is uncertain [13]. Social factors negatively associated with access to rehabilitation are low level of education, unemployment, and substance abuse [8,11,12,14]. In stroke patients, studies suggest that reduced preinjury functional levels negatively influence the decision to refer to rehabilitation [15,16].

In 2012/2014, we published a quasi-experimental study that evaluated whether early initiation of a continuous care and rehabilitation pathway could improve functional outcomes and reduce hospitalization costs for patients with severe TBI [3,17]. We noted that patients with a continuous pathway through treatment had better functional outcomes 12 months postinjury. Across a 5-year period, TBI-related hospitalization costs were reduced, including those for inpatient rehabilitation, and improved outcomes were observed for the patients (under reasonable assumptions) [3,17]. Despite this knowledge, there is a concern that a significant number of patients are still not included in the direct care pathway. Furthermore, hospitals and patient populations are dynamic; thus, there is a constant need for evaluating clinical practice. The study aims to provide an updated overview of discharge to rehabilitation following acute traumatic intracranial injury over a 5-year period (2015–2019) and identify factors associated with a direct pathway to rehabilitation from acute care units.

## 2. Materials and Methods

### 2.1. Study Setting and Participants

Oslo University Hospital (OUH) is the only Level 1 trauma center with neurosurgical services in the southeastern region of Norway, serving 3.0 million inhabitants. OUH also serves as the primary trauma referral hospital for Oslo residents (population ≈ 700,000). Trauma care in Norway is organized through public hospitals with an equal access policy and is free of charge. In 2007, OUH established early specialized rehabilitation and a continuous chain of treatment for severe TBI.

Data were retrieved from the Oslo TBI Registry—Neurosurgery, a quality control database maintained by the neurosurgical department at OUH since 2015. The registration is prospective; data are derived manually from electronic medical records and stored in a Medinsight database [18]. The inclusion criteria for the Oslo TBI Registry—Neurosurgery were (i) traumatic brain injury; (ii) cerebral CT/CTA or cerebral MRI/MRA with findings of acute trauma (hemorrhage, fracture, traumatic axonal injury, vascular injury); (iii) admission to OUH within seven days postinjury; and (iv) Norwegian social security number. A more thorough description of patients in this registry has been published previously [19]. For this study, we included adult patients (age ≥18 years) who were residents of the southeastern region, admitted to OUH between 1 January 2015 and 31 December 2019, and discharged alive from the acute care units at OUH.

### 2.2. Endpoint

The endpoint in this study is discharge destination from the acute care units at OUH. Acute care units are defined as ICUs and surgical wards. Discharge destinations are categorized as home, specialized rehabilitation, local hospital, general rehabilitation, nursing home and other. The endpoint variable was binary: direct transfer to rehabilitation or not. Only patients discharged to specialized inpatient rehabilitation were categorized as “yes”. All other discharge places are considered as “no”. Information regarding inpatient rehabilitation at later stages of TBI was not available, thus not addressed in this study.

### 2.3. Independent Variables

#### 2.3.1. Demographics and Preinjury Comorbidity

Age (stratified into 18–29 years, 30–49 years, 50–64 years, 65–79 years, 80+ years), sex, living status at the time of injury (home independent, home with assistance, or institution (e.g., nursing home)), preinjury comorbidity (as classified by the American Society of Anesthesiologists Physical Status Classification System (ASA-PS)) [20], and any preinjury substance dependence (including alcohol and/or drugs).

#### 2.3.2. Injury Characteristics

Injury characteristics included the trauma mechanism (classified as falls, road traffic accidents (RTAs), other), whether high-energy trauma (defined as falls from a height ≥3 m, RTAs, or other high-energy accidents) was involved, the Glasgow Coma Scale score (GCS) (utilized lowest score documented prior to intubation or admission OUH), TBI severity according to the Head Injury Severity Score (HISS) (minimal: GCS 15 and no loss of consciousness or amnesia; mild: GCS 14 or 15 plus amnesia, or brief loss of consciousness (<5 min), or impaired alertness or memory; moderate: GCS 9–13 or loss of consciousness ≥5 min or focal neurological deficit; or severe: GCS ≤ 8) [21,22]. Computed tomography (CT) findings (primary CT head scan performed at OUH) and magnetic resonance imaging (MRI) (signs of traumatic axonal injury (TAI)) results were also collected. Minimal and mild TBI with traumatic findings on CT is referred to as complicated mild TBI [23,24].

#### 2.3.3. Acute Treatment

Acute treatment involved the following: insertion of intracranial pressure (ICP) sensors and neurosurgical procedures including evacuation of the mass lesion (hematoma/hemorrhage), cerebrospinal fluid drainage, decompressive hemicraniectomy, repair of the dura or fractured skull (duraplasty/cranioplasty) and vascular surgery. Admission to the intensive care unit (ICU) included all patients admitted to the ICU, whereas uncomplicated short stays (<24 h) for TBI observation in the intermediate/step-down unit were registered as ward admissions. Calculation of length of stay (LOS) and days on ventilator were based on dates, with each date counted as a full day.

#### 2.3.4. Functional Outcome

The Glasgow Outcome Score (GOS) on the day of discharge was estimated based on information from multidisciplinary medical records. The GOS is divided into 5 categories: GOS 1 dead (D), GOS 2 vegetative state (VS), GOS 3 severe disability (SD), GOS 4 moderate disability (MD) or GOS 5 good recovery (GR) [25], and only 2 through 5 were applicable in the present study population. The reasons for reduced GOSs were grouped into (i) TBI, (ii) TBI in combination with extracranial injury and/or comorbidity, and (iii) other.

### 2.4. Statistics

Patient characteristics are reported as frequencies (percentages) and means (standard deviations) or medians (interquartile ranges) depending on the distribution. We divided patients into two groups based on the endpoint variable. For comparisons between groups, we used *t*-tests or Mann–Whitney U tests for continuous variables and χ^2^ tests or Fisher’s exact tests for categorical variables, as appropriate. All tests were two-sided and used the 5% significance level. To examine whether the proportion of patients with moderate–severe TBI who followed the direct pathway increased over the years, logistic regression analysis was used. In the trend analysis, the year variable was treated as an ordinal score. Univariate and multivariate logistic regressions were run to examine independent predictors differentiating between patients discharged to specialized rehabilitation and patients discharged elsewhere. The first model included all patients, and the second model was a subgroup analysis that included patients with moderate–severe TBI. Independent variables were selected based on previous literature and clinical importance. Before conducting the multiple regression analysis, possible multicollinearity of the independent variables was examined. ICU LOS correlated with ICP sensor (r > 0.68), and GCS at discharge was correlated with GOS (r > 0.66); thus, ICU LOS and GCS were not included in the models. Patients with GOS 5-GR were not eligible for inpatient rehabilitation and hence were not included in the models. Evaluation of the predictive accuracy of the models was assessed by calibration and discrimination. Calibration was evaluated by the Hosmer and Lemeshow goodness-of-fit test, and a statistically nonsignificant result (*p* > 0.05) suggests that the model predicts accurately on average. Discrimination was evaluated by analysis of the area under the receiver operating characteristic curve (AUC ROC). We defined acceptable discriminatory capability as an AUC ROC greater than 0.7 [26]. The results are presented with odds ratios (ORs), 95% confidence intervals (CIs) and *p*-values. Data were analyzed with IBM SPSS Statistics, Version 26.0. Armonk, NY, USA: IBM Corp.

### 2.5. Ethics

The OUH data protection officer (DPO) approved the Medinsight database (approval number 2016/17569) and this study (approval number 18/20658).

## 3. Results

A total of 1887 patients ≥18 years, residents of the region and with CT-verified TBI were admitted to OUH from 2015 to 2019. Patients discharged as dead (GOS 1) were excluded (*n* = 163); thus, 1724 patients discharged alive from the acute care units at OUH were included in this study. The mean patient age was 57 years (SD 20), 69% were males, 87% lived independently at home, and 69% had a preinjury ASA-PS score of 1–2. The most frequent trauma mechanism was falls (56%). Head injury severity was categorized as complicated mild in 49%, moderate in 30% and severe in 21%. Further patient and injury characteristics are given in Table 1.

ICU admission was registered for 64% of the patients, with a median ICU stay of 3 days (IQR 2–10). A neurosurgical procedure was performed in 21% of patients, evacuation of mass lesions was the most frequently performed procedure (13%). An ICP sensor was inserted in 22%. Data on acute treatments provided are presented in Table 2.

Functional outcomes at the time of discharge from the acute care units, measured with the GOS, indicated good recovery in 5%, moderate disability in 46%, severe disability in 46%, and a vegetative state in 3% (Table 3). In patients with good recovery as measured by the GOS, 82/83 (98.8%) were discharged directly home (one patient was discharged to “other”). The two main reasons registered for reduced functional outcome (GOS < 5) were TBI alone and a combination of TBI/extracranial injury/comorbidity. The majority of patients were discharged to their local hospital (43%), followed by home (32%) and specialized rehabilitation (17%) (Table 3). In patients with severe TBI, 39% (139/353) entered direct pathway, and 20% (105/520) of patients with moderate TBI.

Table 1, Table 2 and Table 3 contain a comparison of patients discharged to specialized rehabilitation (direct pathway group) with patients discharged to other destinations (indirect pathway group). Comparing the two groups, the direct pathway group was younger, was more often male, had less comorbidity, had more severe TBI, had more intensive hospital treatment (neurosurgical procedures, ICP monitoring, ICU stay and days on ventilator), and had lower GOSs at the time of discharge.

**Figure 1:**
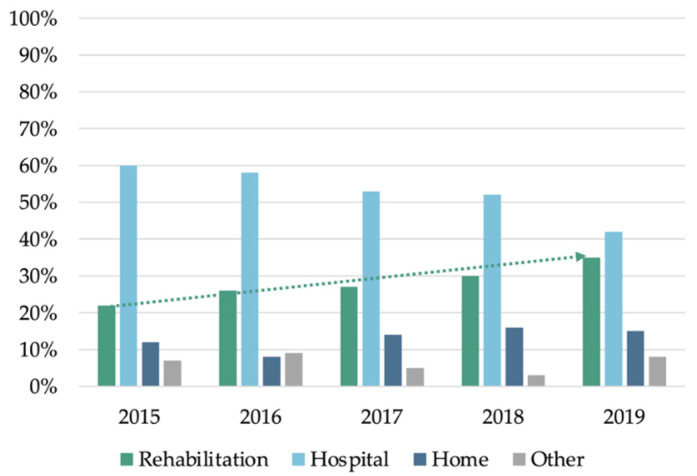
Direct pathway by year for patients with moderate–severe TBI (*n* = 873). The percentage of patients following the direct pathway (rehabilitation) increased during the period. “Other” includes general rehabilitation, nursing home and other.

The proportion of patients with moderate–severe TBI (N = 873) discharged directly to rehabilitation increased during the period, from 22% (36/166) in 2015 to 35% (63/180) in 2019 (Figure 1). A patient with moderate–severe TBI admitted in 2019 had higher odds for entering a direct pathway than a patient admitted in 2015 (OR 1.17, CI 1.05–2.30, ptrend = 0.004).

**Figure 2:**
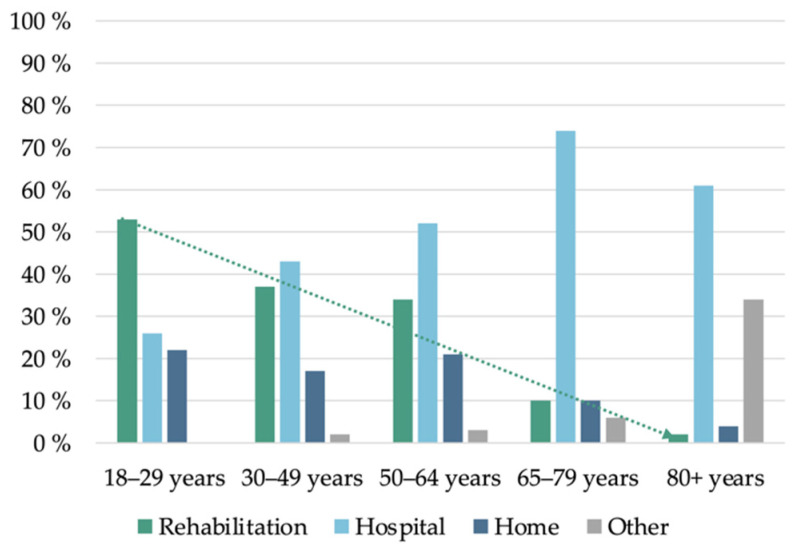
Direct pathway by age strata for patients with moderate–severe TBI (*n* = 873). The percentage following the direct pathway (rehabilitation) decreased with increasing age. “Other” includes general rehabilitation, nursing home and other.

However, the proportions of direct pathways to rehabilitation differed substantially between age strata (moderate–severe TBI). For the youngest patient stratum (18–29 years), the proportion following the direct pathway was 53%, in contrast to patients of retirement age (65–79 years) in which it dropped to 10%, with the majority discharged to local hospitals (74%, 166/225). The distribution of discharge locations within age strata is shown in Figure 2.

To identify potential predictors for discharge directly to a rehabilitation unit, uni- and multi-variate logistic regression was performed. In univariate logistic regression, the following factors were associated with an increased likelihood of discharge directly to a rehabilitation unit: younger age, male sex, living independently, low preinjury comorbidity (ASA 1–2), increased TBI severity, placement of ICP sensors, neurosurgical procedures, extracranial surgery, lower GOS at the time of discharge, and lower GOS at discharge due to TBI and no concomitant extracranial injury. Substance dependence showed no association with direct transfer to specialized rehabilitation and hence not included in the multivariate models. In multivariate regression, the following factors remained significantly associated with an increased likelihood of discharge directly to a rehabilitation unit: younger age, living independently, low preinjury comorbidity (ASA 1–2), increased TBI severity, lower GOS at the time of discharge, and lower GOS at discharge due to TBI and no concomitant extracranial injury (Table 4). Subgroup analysis for patients with moderate–severe TBI showed similar results, except lower GOS due to TBI was not significantly associated with an increased likelihood of entering a direct pathway (Table 5).

## 4. Discussion

### 4.1. Main Findings

In this study, we aimed to provide an overview of discharge to specialized rehabilitation following acute TBI from 2015 to 2019 and to identify factors associated with a direct pathway to rehabilitation from acute care units. We found a significant positive trend in the number of patients who followed the direct pathway during the five-year study period. Patients discharged to the direct pathway typically had the following characteristics: younger age, low preinjury comorbidity, moderate–severe TBI and disability due TBI at the time of discharge. However, the study revealed significant differences in the proportions of patients following a direct pathway among age strata.

### 4.2. Patient Characteristics and Direct Pathway

The study population is similar to the western TBI population in terms of the proportion of males (69%), age (mean 57 years) and the dominant trauma mechanism being falls (56%) [27,28]. Severe somatic comorbidity was found in 29% (ASA-PS 3), which is higher than in the CENTER-TBI case-mix study, which also included patients with concussions (11%) [28]. This can likely be explained by a somewhat higher mean age in our study population and no exclusion criteria based on preinjury disease. This is also reflected by the 13% of patients living with assistance at home or at a nursing home at the time of injury. We found preinjury substance dependence in 17% of patients. However, patients were not systematically assessed for substance dependence; thus, there is reason to believe the actual number is somewhat higher. By comparison, a previous study from OUH found that 26% of TBI patients had significant preinjury substance dependence (mainly alcohol) [29].

Half of the patient population was admitted with complicated mild TBI, and one-third was discharged to home. In line with previous research, patients with moderate–severe TBI dominated in the direct pathway group [11,12]. For patients with severe TBI, 39% followed the direct pathway, which was slightly lower than that in previous European studies (40–48%) [8,12,14,30]. Similar to the study by de Koning et al. [30], 20% of moderate TBI followed the direct pathway. The results from the CENTER-TBI study demonstrated different and complex care pathways in the first six months after injury, particularly for patients with severe TBI [7]. Furthermore, rehabilitation needs were reported in 90% of patients with moderate–severe TBI in the first six months after injury [31]. Our data are limited to acute treatment at a Level 1 trauma hospital, and it is likely that several patients were referred to specialized rehabilitation at a later point. However, there are different organizations and expertise in rehabilitation between local hospitals and municipalities within the health region. Thus, it is reasonable to assume that the probability of unmet rehabilitation needs increases when the direct pathway is broken.

We found a positive and significant trend for utility of the direct pathway during the five years–a result of emphasized focus on TBI rehabilitation during the past decade [3,17]. However, the situation is fragile, and it is worth mentioning that neurorehabilitation beds were periodically converted to manage the impact of COVID-19 at OUH. However, this study did not include patients injured in the 2020–2021 pandemic years. Results on the access to rehabilitation in the pandemic years will be published in a subsequent paper.

### 4.3. Factors Predicting the Direct Pathway

The probability of following the direct pathway increased for patients with more severe TBI, younger age, and decreased functional level at discharge as measured by the GOS. These findings are in line with previous research [13,32]. The statistical models demonstrated the striking impact of age, which is discussed in a separate paragraph. Neurosurgical procedures were significant predictors in univariate regression but not in multivariate models, which is inconsistent with the study by Jacob et al. [11]. In fact, 42% of patients following the direct pathway were treated without surgical procedures. Severe disability (GOS) was the strongest clinical positive predictor in both models, suggesting that patients are clinically assessed and prioritized for the direct pathway. In this study, TBI severity was categorized by HISS, which is mainly based on the GCS score in the acute phase. The GCS score is essential because it partly guides acute treatment [22,33,34]. The GCS score, as a measure of TBI severity, is widely used in both clinical settings and research and correlates with outcome at the group level, but it should not be used as a single injury severity predictor of TBI outcome [34]. TBI is a complex condition with substantial individual variation in outcomes. TBI can be life-threatening in the acute phase, e.g., in cases with epidural hematoma, where the patient may have rapid and good recovery if treated with immediate neurosurgery and removal of hematoma. Likewise, patients with moderate or mild TBI in the acute phase may experience long-term disability. A recent proposal in assessing the severity of TBI suggests changing from severity labels to risk assessment over time [35]. Reduced functional levels at six months are reported for patients with TBI admitted to the hospital [28]. The study identifying unmet rehabilitation needs emphasized the necessity of a more extensive and standardized assessment of functional impairments and corresponding rehabilitation needs [31]. Currently, there is no systematic assessment of rehabilitation needs at discharge from acute care units at OUH. Moreover, the decision for referral transfer to rehabilitation is not solely based on the clinical condition of patients. It can also be affected by pressure to free beds at the Level 1 trauma center, low capacity at early rehabilitation units, and professionals’ knowledge about expected benefit from rehabilitation and long-term disabilities associated with TBI [12,36,37].

We found no support for the notion that patients with preinjury substance dependence are downgraded for the direct pathway, which was in contrast to findings by Jourdan et al. [12] but in line with other studies [11,13,14]. In our study, access to sociodemographic variables was limited. However, we do not believe that such variables have had an impact on the direct pathway in this study; this assumption is based on results regarding preinjury substance dependence. Nonetheless, variables such as marital status, education and employment are expected to be of importance at later stages of TBI when follow-up is more fragmented.

### 4.4. The Impact of Age

Age was an important explanatory variable for the direct pathway. The probability of management through the direct pathway decreased significantly with higher age, a situation not unique to our study population. In a systematic review [13], the only consistent negative predictor for discharge to specialized rehabilitation was increasing age. Preinjury comorbidity and functional impairments were highly associated with age in our study population [19]. We found these factors to have a significant negative impact on the probability of treatment in the direct pathway, similar to the literature on stroke [15,16]. Previous studies show that younger patients to a greater extent follow a direct pathway or are discharged to home, while older patients are more often discharged to general rehabilitation and rarely directly to home [13,14,38,39]. Clinical trials typically include patients aged 18–65 years and often have exclusion criteria based on comorbidities [28,40,41]. Older patients receive less aggressive therapy in the acute phase (medical and surgical) [42,43]; presumably, this may lead to directions for further treatment. Nonetheless, there is evidence that older patients with TBI may benefit from intensive inpatient rehabilitation [41]. It is clear from our results that young adults and patients of working age are prioritized for the direct pathway. Given the risk of life-long negative consequences of TBI, one can argue that it is appropriate to prioritize these patients. However, life expectancy is increasing, and in society, we observe many persons >64 years living an active life with social roles that include responsibilities with indirect socioeconomic impact (e.g., voluntary work, family obligations across generations). Moreover, it will presumably be socioeconomically beneficial if older patients regain preinjury functional levels and are able to live independently at home.

### 4.5. Strengths, Limitations and Future Directions

The strength of this study is the inclusion of a real-world TBI population from a defined geographical region with little migration and no exclusion criteria based on age or preinjury comorbidities. The study provides a useful overview and captures trends regarding patient flow into the direct pathway to specialized rehabilitation from a Level 1 trauma center. However, there are limitations to consider. There was no information available in the Oslo TBI Registry—Neurosurgery on later access to rehabilitation for patients not included in the direct pathway. In addition, there was a lack of information on longer-term outcomes for both groups. Thus, we cannot draw firm conclusions on the impact of direct or indirect care pathways in this study. Furthermore, the variables are crude and based on acute clinical parameters; they do not explain the multifaceted reality at the individual level. Information in the database is derived from medical records, where it is well known that information quality is variable. Moreover, database coding errors cannot be completely ruled out, although the database is continuously searched and adjusted for coding errors.

Our results highlight the importance of continued focus on optimizing and maintaining a direct TBI care pathway and systematic assessment of rehabilitation needs during the acute phase for all hospital-admitted patients with TBI. To do so, it would be beneficial to develop recommendations for clinical practice to assess rehabilitation needs before patients are discharged from acute care. Finally, future studies on TBI care pathways should focus on patients >64 years.

## Figures and Tables

**Table 1:** Patient and injury characteristics.

	All Patients	Direct Pathway ^1^	Indirect Pathway ^2^	*p*-Value
	*n* = 1724 (100%)	*n* = 289 (100%)	*n* = 1435 (100%)	
Age				<0.001
In years, mean (SD)	57 (20)	45 (17)	59 (20)	
Strata				
18–29	233 (13.5)	75 (26.0)	158 (11.0)	
30–49	356 (20.6)	82 (28.4)	274 (19.1)	
50–64	438 (25.4)	94 (32.5)	344 (24.0)	
65–79	449 (26.0)	35 (12.1)	414 (28.9)	
80+	248 (14.4)	3 (1.0)	245 (17.1)	
Male	1189 (69.0)	223 (77.2)	966 (67.3)	0.001
Living status				<0.001
Home independent	1493 (86.6)	281 (97.2)	1212 (84.5)	
Home assisted	163 (9.5)	5 (1.7)	158 (11.0)	
Nursing home	46 (2.7)	0	46 (3.2)	
Other/unknown	22 (1.3)	3 (1.0)	19 (1.3)	
ASA-PS				<0.001
1 Normal healthy	696 (40.4)	170 (58.8)	526 (36.7)	
2 Mild systemic	500 (29.0)	75 (26.0)	425 (29.6)	
3 Severe systemic	506 (29.4)	44 (15.2)	421 (32.2)	
4 Life threatening	22 (1.3)	0	22 (1.5)	
Substance dependence	292 (16.9)	48 (16.6)	244 (17.0)	0.870
Trauma mechanism				<0.001
Fall	968 (56.1)	115 (39.8)	853 (59.4)	
Road traffic	390 (22.6)	93 (32.2)	297 (20.7)	
Other	366 (21.2)	81 (28.0)	285 (19.9)	
Head Injury Severity				<0.001
Minimal	106 (6.1)	1 (0.3)	105 (7.3)	
Mild	745 (43.2)	44 (15.2)	701 (48.9)	
Moderate	520 (30.2)	105 (36.3)	415 (28.9)	
Severe	353 (20.5)	139 (48.1)	214 (14.9)	
Isolated TBI	892 (51.7)	118 (40.8)	774 (53.9)	<0.001
High-energy trauma	614 (35.6)	167 (57.8)	447 (31.1)	<0.001
CT findings ^3^				
Skull fracture ^4^	878 (49.1)	188 (65.1)	690 (48.1)	<0.001
Acute subdural hematoma	958 (55.6)	11 (66.1)	767 (53.4)	<0.001
Epidural hematoma	244 (14.2)	62 (21.5)	182 (12.7)	<0.001
Contusion	837 (48.5)	201 (69.6)	636 (44.3)	<0.001
tSAH ^5^	1021 (59.2)	207 (71.6)	814 (56.7)	<0.001
Midline shift > 5 mm	236 (13.7)	62 (21.5)	174 (12.1)	<0.001
Basal cisterns abnormal	203 (11.8)	72 (24.9)	131 (9.1)	<0.001
MRI performed	459 (26.6)	201 (69.6)	258 (18.0)	<0.001
Traumatic axonal injury	251 (14.6)	133 (46.0)	118 (8.2)	<0.001

^1^ Patient discharged directly to specialized rehabilitation. ^2^ Patient discharged to home, local hospital, nursing home or other. ^3^ One patient may have more than one type of traumatic pathology. ^4^ Includes basilar, linear vault, depressed vault. ^5^ tSAH: traumatic subarachnoidal hemorrhage.

**Table 2:** Acute treatment.

	All Patients	Direct Pathway ^1^	Indirect Pathway ^2^	*p*-Value
	*n* = 1724 (100%)	*n* = 289 (100%)	*n* = 1435 (100%)	
ICU admission	1111 (64.4)	271 (93.8)	840 (58.5)	<0.001
Days in ICU Median (IQR)	3 (2–10)	8 (3–22)	3 (2–6)	<0.001
Intubated	501 (29.1)	179 (61.9)	322 (22.4)	<0.001
Days on ventilator Median (IQR)	6 (2–15)	13 (3–22)	4 (1–11)	<0.001
Neurosurgery ^3^	358 (20.8)	114 (39.4)	244 (17.0)	<0.001
Evacuation mass lesion	231 (13.4)	72 (24.9)	159 (11.1)	<0.001
Decompressive hemicraniectomy	26 (1.5)	18 (6.2)	8 (0.6)	<0.001
CSF diversion	115 (6.7)	58 (20.1)	57 (4.0)	<0.001
Duraplasty/cranioplasty	103 (6.0)	27 (9.3)	76 (5.3)	0.008
Vascular surgery	12 (0.7)	6 (2.1)	6 (0.4)	0.008
ICP-sensor	371 (21.5)	153 (52.9)	218 (15.2)	<0.001
Extracranial surgery	375 (21.8)	96 (33.2)	279 (19.4)	<0.001

^1^ Patient discharged directly to specialized rehabilitation. ^2^ Patient discharged to home, local hospital, nursing home or other. ^3^ Neurosurgery includes evacuation of mass lesions, decompressive hemicraniectomy, CSF diversion, duraplasty/cranioplasty, and vascular surgery. One patient may undergo several procedures.

**Table 3:** Day of discharge from acute care units at OUH: functional level and destination.

	All Patients	Direct Pathway ^1^	Indirect Pathway ^2^	*p*-Value
	*n* = 1724 (100%)	*n* = 289 (100%)	*n* = 1435 (100%)	
GCS 15	1136 (65.9)	128 (44.3)	1008 (70.2)	<0.001
Ventilator-dependent	125 (7.3)	1 (0.3)	124 (8.6)	---
GOS ^3^				<0.001
Vegetative State	47 (2.7)	6 (2.1)	41 (2.9)	
Severe Disability	799 (46.3)	233 (80.6)	566 (39.4)	
Moderate Disability	791 (45.9)	50 (17.3)	741 (51.6)	
Good Recovery	83 (4.8)	0	83 (5.8)	
Not available	4 (0.3)	0	4 (0.3)	
Reduced GOS reason TBI TBI + extracranial injury/comorbidity Other	877 (50.9)	198 (68.5)	679 (47.3)	<0.001
	606 (35.2)	76 (26.3)	530 (36.9)	
	241 (14.0)	15 (5.2)	226 (15.7)	
Discharge to		289 (100)		---
Home	554 (32.1)		554 (38.6)	
Local hospital	745 (43.3)		745 (51.9)	
Specialized rehabilitation	289 (16.8)		---	
General rehabilitation	10 (0.6)		10 (0.7)	
Nursing home	105 (6.1)		105 (7.3)	
Other	21 (1.2)		21 (1.5)	

^1^ Patient discharged directly to specialized rehabilitation. ^2^ Patient discharged to home, local hospital, nursing home or other. ^3^ GOS: Glasgow Outcome Score.

**Table 4:** Predictors associated with discharge directly to a rehabilitation unit (*n* = 1724). A total of 1637 patients were included in the model; 87 were excluded (83 patients’ GOS 5-GR, 4 patients’ GOS not available).

	Univariate			Multivariate Model ^1^		
Variables	OR	95% CI	*p*-Value	OR	95% CI	*p*-Value
Age strata						
18–29	1			1		
30–49	0.63	0.44, 0.92	0.017	0.51	0.33, 0.81	0.004
50–64	0.56	0.39, 0.81	0.002	0.44	0.28, 0.69	<0.001
65–79	0.19	0.12, 0.30	<0.001	0.15	0.09, 0.26	<0.001
80+	0.03	0.01, 0.08	<0.001	0.04	0.01, 0.13	<0.001
Sex						
Female	1			1		
Male	1.65	1.22, 2.20	0.001	1.26	0.88, 1.80	0.213
ASA						
1–2	1			1		
3–4	0.34	0.24, 0.48	<0.001	0.59	0.39, 0.90	0.014
Living status: independent						
No	1			1		
Yes	6.81	3.32, 13.96	<0.001	3.98	1.79, 8.86	0.001
HISS						
Mild	1			1		
Moderate	4.18	2.89, 6.04	<0.001	2.20	1.43, 3.39	<0.001
Severe	10.74	7.42, 15.54	<0.001	3.19	1.91, 5.32	<0.001
Neurosurgery						
No	1			1		
Yes	2.96	2.25, 3.90	<0.001	1.09	0.73, 1.61	0.682
ICP sensor						
No	1			1		
Yes	5.93	4.51, 7.79	<0.001	1.19	0.76, 1.86	0.446
Extracranial surgery						
No	1			1		
Yes	1.96	1.48, 2.59	<0.001	1.30	0.85, 1.97	0.227
GOS at discharge						
MD	1			1		
SD	6.10	4.41, 8.44	<0.001	6.78	4.39, 10.47	<0.001
VS	2.17	0.88, 5.35	0.093	1.50	0.52, 4.31	0.451
Reason for reduced GOS						
Other	1			1		
TBI	2.92	1.68, 5.09	<0.001	2.25	1.07, 4.72	0.032
TBI + extracranial injury/comorbidity	1.44	0.80, 2.57	0.224	1.30	0.65, 2.60	0.459

^1^ The Hosmer and Lemeshow goodness-of-fit test was not significant, indicating a satisfactory fit of the model (χ 2 = 3.05, df = 8, *p* = 0.93). The area under the ROC curve was 0.86 (95% CI: 0.84–0.88), indicating good discriminative ability.

**Table 5:** Predictors associated with discharge directly to a rehabilitation unit. Subgroup analysis of patients admitted with moderate–severe TBI (*n* = 873): 862 patients were included in the model; 11 were excluded (7 patients’ GOS 5-GR, 4 patients’ GOS not available).

	Univariate			Multivariate Model ^1^		
Variables	OR	95% CI	*p*-Value	OR	95% CI	*p*-Value
Age strata						
18–29	1			1		
30–49	0.63	0.44, 0.92	0.017	0.51	0.33, 0.81	0.004
50–64	0.56	0.39, 0.81	0.002	0.44	0.28, 0.69	<0.001
65–79	0.19	0.12, 0.30	<0.001	0.15	0.09, 0.26	<0.001
80+	0.03	0.01, 0.08	<0.001	0.04	0.01, 0.13	<0.001
Sex						
Female	1			1		
Male	1.65	1.22, 2.20	0.001	1.26	0.88, 1.80	0.213
**ASA**						
1–2	1			1		
3–4	0.34	0.24, 0.48	<0.001	0.59	0.39, 0.90	0.014
Living status: independent						
No	1			1		
Yes	6.81	3.32, 13.96	<0.001	3.98	1.79, 8.86	0.001
HISS						
Mild	1			1		
Moderate	4.18	2.89, 6.04	<0.001	2.20	1.43, 3.39	<0.001
Severe	10.74	7.42, 15.54	<0.001	3.19	1.91, 5.32	<0.001
Neurosurgery						
No	1			1		
Yes	2.96	2.25, 3.90	<0.001	1.09	0.73, 1.61	0.682
ICP sensor						
No	1			1		
Yes	5.93	4.51, 7.79	<0.001	1.19	0.76, 1.86	0.446
Extracranial surgery						
No	1			1		
Yes	1.96	1.48, 2.59	<0.001	1.30	0.85, 1.97	0.227
GOS at discharge						
MD	1			1		
SD	6.10	4.41, 8.44	<0.001	6.78	4.39, 10.47	<0.001
VS	2.17	0.88, 5.35	0.093	1.50	0.52, 4.31	0.451
Reason for reduced GOS						
Other	1			1		
TBI	2.92	1.68, 5.09	<0.001	2.25	1.07, 4.72	0.032
TBI + extracranial injury/comorbidity	1.44	0.80, 2.57	0.224	1.30	0.65, 2.60	0.459

^1^ The Hosmer and Lemeshow goodness-of-fit test was not significant, indicating satisfactory fit of the model (χ^2^ = 4.14, df = 8, *p* = 0.85). The area under the ROC curve was 0.82 (95% CI: 0.80–0.85), indicating good discriminative ability.

## Data Availability

The data presented in this study are available on request from the corresponding author if considered appropriate. The data are not publicly available due to privacy and ethical restrictions.
